# Probiotics for vaginal health in South Africa: what is on retailers’ shelves?

**DOI:** 10.1186/s12905-017-0362-6

**Published:** 2017-01-19

**Authors:** Anna-Ursula Happel, Shameem Z. Jaumdally, Tanya Pidwell, Tracy Cornelius, Heather B. Jaspan, Remy Froissart, Shaun L. Barnabas, Jo-Ann S. Passmore

**Affiliations:** 1 Institute of Infectious Disease and Molecular Medicine, University of Cape Town, Medical School, Anzio Road, Observatory, 7925 Cape Town, South Africa; 2 UMR 5290 MIVEGEC, CNRS IRD Université Montpellier, Montpellier, France; 3 CAPRISA DST-NRF Centre of Excellence in HIV Prevention, Cape Town, South Africa; 4 Desmond Tutu HIV Foundation, Cape Town, South Africa; 5 Seattle Children’s Research Institute, University of Washington, Seattle, WA USA; 6 National Health Laboratory Service, Cape Town, South Africa

**Keywords:** Probiotics, Women, Vaginal, *Lactobacillus* spp.

## Abstract

**Background:**

Probiotics are widely used to improve gastrointestinal (GI) health, but they may also be useful to prevent or treat gynaecological disorders, including bacterial vaginosis (BV) and candidiasis. BV prevalence is high in South Africa and is associated with increased HIV risk and pregnancy complications. We aimed to assess the availability of probiotics for vaginal health in retail stores (pharmacies, supermarkets and health stores) in two major cities in South Africa.

**Methods:**

A two-stage cluster sampling strategy was used in the Durban and Cape Town metropoles. Instructions for use, microbial composition, dose, storage and manufacturers’ details were recorded.

**Results:**

A total of 104 unique probiotics were identified in Cape Town and Durban (66.4% manufactured locally). Cape Town had more products than Durban (94 versus 59 probiotics), although 47% were common between cities (49/104). Only four products were explicitly for vaginal health. The remainder were for GI health in adults (51.0%) or infants (17.3%). The predominant species seen overall included *Lactobacillus acidophilus* (53.5%), *L. rhamnosus* (37.6%), *Bifidobacterium longum ssp. longum* (35.6%) and *B. animalis ssp. lactis* (33.7%). Products for vaginal health contained only common GI probiotic species, with a combination of *L. acidophilus/B. longum ssp. longum/B. bifidum*, *L. rhamnosus/L. reuteri* or *L. rhamnosus* alone, despite *L. crispatus*, *L. gasseri,* and *L. jensenii* being the most common commensals found in the lower female reproductive tract.

**Conclusion:**

This survey highlights the paucity of vaginal probiotics available in South Africa, where vaginal dysbiosis is common. Most vaginal products contained organisms other than female genital tract commensals.

**Electronic supplementary material:**

The online version of this article (doi:10.1186/s12905-017-0362-6) contains supplementary material, which is available to authorized users.

## Background

Maintenance of vaginal health is thought to play an important role in protecting women from reproductive complications and the acquisition of sexually transmitted infections (STIs) [1–10]. A healthy vagina is generally thought to be defined as one with a low pH and a *Lactobacillus* spp. dominance. Commensal *Lactobacillus* spp. in the genital tract have been thought to contribute to genital health by their ability to lower vaginal pH, to produce bactericidal compounds, and to competitively outcompete potentially pathogenic bacteria [8, 11–14].

A healthy lower female reproductive tract is dominated by various *Lactobacillus* spp., with *L. crispatus, L. gasseri, L. jensenii, L. iners* and *L. vaginalis* the most frequent and abundant organisms present in North American women [8, 9]. Several studies have shown that the predominant bacterial species colonizing the female genital tract differ by geography and ethnicity [8]. Only 37% of asymptomatic South African women had a *Lactobacillus* spp. dominant vaginal microbiome compared to 90% of white and 62% of black women in developed countries [8, 15, 16]. Within North American women, *Lactobacillus* predominance differed by ethnic group, with 89.7% of white and 80.2% of Asian women having a genital tract dominated by *L. crispatus, L. gasseri, L. iners* and *L. jensenii.* Only 61.9% of black and 59.6% of Hispanic women showed a similar pattern [8]. In South Africa the predominant *Lactobacillus* spp. found in young women in KwaZulu Natal was *L. iners*, which differs from the species described in the female genital tracts of North American women [15].

Depletion of commensal *Lactobacillus* spp., an increase in bacterial diversity and an overgrowth of pathogenic bacteria are associated with the development of bacterial vaginosis (BV) [17]. Although not considered a STI, BV is the most prevalent condition to influence vaginal health in women of reproductive age, and is considered to be enhanced by sexual activity [18]. It increases risk of endometritis, preterm delivery, chorioamnionitis, spontaneous abortion, maternal/neonatal sepsis [1–3, 19–21] and susceptibility to viral (including HIV, HPV and HSV-2) or bacterial (including *Trichomonas vaginalis, Neisseria gonorrhoeae* and *Chlamydia trachomatis*) infections [4–7, 22, 23]. Its prevalence varies both between and within countries and is thought to be influenced by host genetics and social factors [24]. The prevalence is the lowest in Asia and Europe (4.5 to 24%) and highest in Sub-Saharan Africa (SSA; 6 to 58%) [25]. The high rates in SSA and the association with a more than three-fold increase in HIV transmission in a region bearing the burden of HIV underlines the urgent need for effective treatment [26].

Despite these serious adverse outcomes, few effective strategies to manage BV and its recurrence exist. Identifying women with BV is challenging as most cases are asymptomatic [27, 28]. Furthermore, antibiotic treatment of BV, the current standard of care, results in only a temporary decrease in dysbiosis with high recurrence rates. Approximately 30% of cases recur within three months of treatment and 50% within six months [29]. Studies that investigated the efficacy of probiotics in the treatment of BV have mostly reported improved cure and no adverse events [30, 31]. However, there is substantial heterogeneity between these trials, with differences in bacterial species and strains used, dose and duration of treatment, route of administration, and population studied.

Probiotics are defined as “live microorganisms, that, when administered in adequate amounts, confer a health benefit on the host” [32]. The bacteria typically contained in probiotics include a diverse spectrum of *Lactobacillus* spp. (including *L. rhamnosus, L. casei, L. acidophilus* and *L. plantarum*) and *Bifidobacterium* spp. (including *B. breve, B. bifidum, B. infantis and B. animalis*), which colonize the healthy human gastrointestinal tract (GIT) [33]. Additionally, yeast strains such as *Saccharomyces cerevisiae* or *S. boulardii* are sometimes included into formulation [33]. Probiotics have been used to treat or prevent disorders of the GIT and immune system in both adults and infants [34, 35]. Probiotics are also used to maintain vaginal health, including for the treatment of BV [30]. In order for the probiotic bacteria to positively impact vaginal health, they first need to colonize the female genital tract successfully. For a successful colonization and the ability to confer a health benefit to the host, the bacteria need to fulfil various criteria including: adherence to vaginal epithelial cells, production of hydrogen peroxide, bacteriocins and biosurfactant, restoration of vaginal pH, and inhibition of potential pathogens associated with BV [36].

Probiotics for vaginal health have been administered vaginally and orally. Oral probiotics were first considered in 2001, when it was shown that probiotic bacteria can passively move from the rectum to the female genital tract [37, 38]. Oral administration of a two strain combination of *L. rhamnosus* GR-1® and *L. reuteri* RC-14® was shown to increase the level of those species in stool and in the vagina, supporting the notion of ano-vaginal transfer [39]. An oral daily dose of over one billion colony-forming units (cfu) maintained a *Lactobacillus*-dominated vaginal microbiome [37]. Additionally, it has been suggested that oral probiotics may inhibit the ano-vaginal transfer of yeast and pathogenic bacteria [40]. However, the level of probiotic bacteria delivered by oral administration is lower than following vaginal administration [41]. Importantly, the time required to affect vaginal health is longer with oral than direct vaginal administration and depends on the viability of the bacteria after they pass through the GIT [39].

Probiotics are often administered along with prebiotics (then called synbiotics) as this is thought to selectively support the growth of probiotic microbiota, thereby increasing their persistence [42, 43]. Commonly used prebiotics are short-chain carbohydrates, particularly inulin, oligosaccharides and pyrodextrins, which are resistant to digestion, fermentable by intestinal microbes and support the selective growth of administered bacteria [33].

The global probiotic market was reported to be USD 27.9 billion in 2011, with an estimated annual growth of 6.8% (http://www.nutraingredients.com/Markets-and-Trends/Global-probiotics-market-to-grow-6.8-annually-until-2018). This rapid growth has created the need for effective legislative regulations of probiotics. The United States Food and Drug Administration (FDA) states that probiotics may be regulated as dietary supplements, conventional food or meal replacement, or as drugs depending on their intended use (http://www.fda.gov/regulatoryinformation/guidances/ucm144657.htm). Similarly, the South African Medicines Control Council (MCC) classifies probiotic-containing products either as complementary medicine or as medicines when making medicinal claims or containing ≥ 1 × 10^9^ cfu per dose unit (http://www.mccza.com/Publications). Despite this, probiotics are not included in the treatment covered by some of the largest medical insurance companies in South Africa (Fedhealth, Bonitas and Momentum).

The aim of this survey was to review probiotics available on the South African retail market and evaluate their likely suitability for the treatment of BV in South African women.

## Methods

### Data collection

**Fig. 1:**
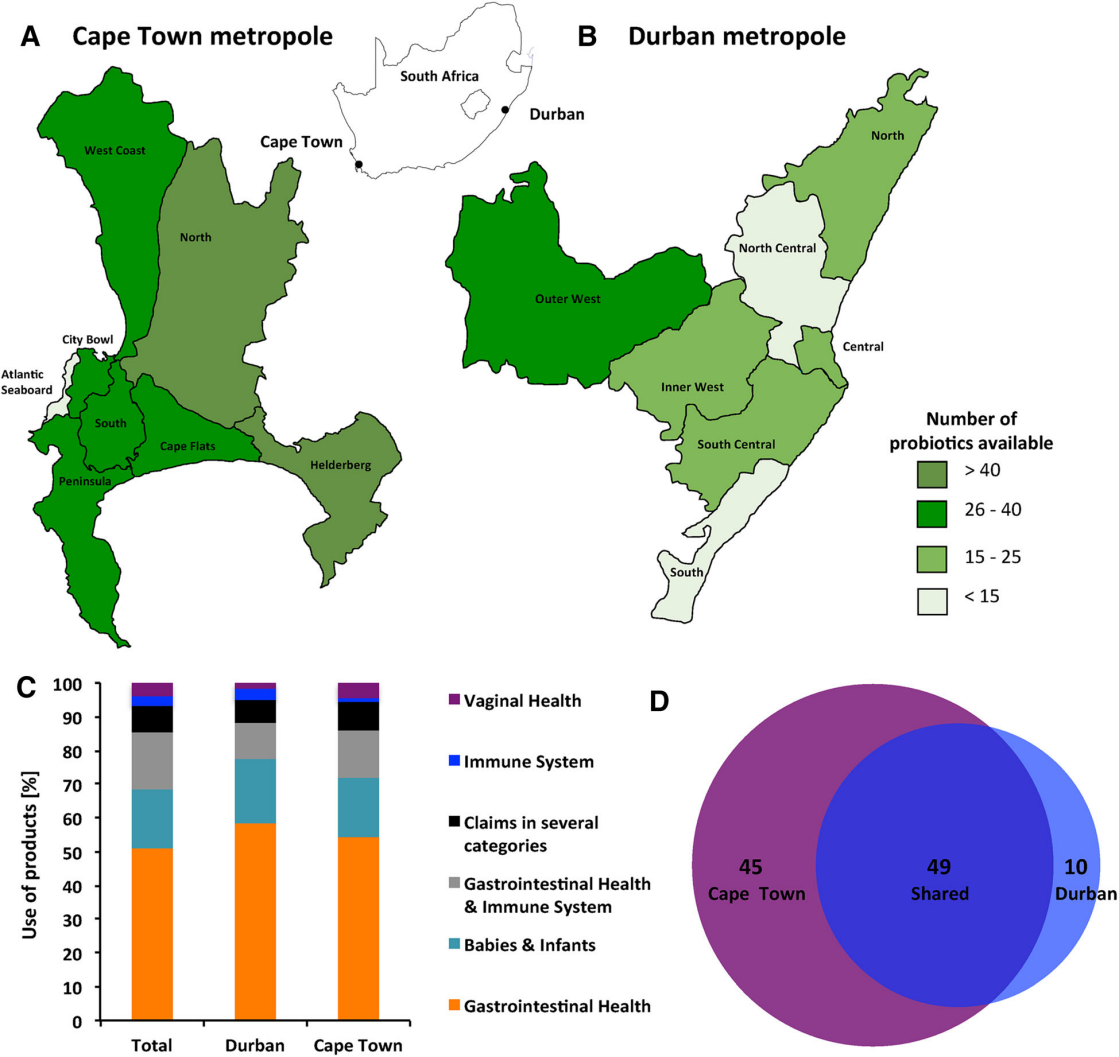
The South African probiotic market. Availability of probiotics by districts in the Cape Town (**a**) and Durban (**b**) metropoles. Cape Town consists of eight districts while Durban metropole is made up of seven districts. A colour scale was used to denote variation in the number of distinct probiotics available for each district. **c** Usage of probiotics. Each product was grouped into one of six categories according to their health claim; and the overall or city-specific distribution is depicted by the stacked bars. **d** Market share Cape Town vs. Durban. The area proportional Venn diagram represents the number of probiotics marketed exclusively in Cape Town (*purple*) or Durban (*light blue*) and those common to both cities (*blue*)

A cross-sectional survey by means of a two-stage cluster sampling was conducted in Durban and Cape Town, South Africa, between September 2015 and January 2016. The primary clusters were geographically demarcated and consisted of the seven and eight districts constituting the Durban and Cape Town metropoles, respectively. The secondary clusters were the major shopping malls in each of the respective districts. Details on all probiotic products in every store retailing probiotics, including pharmacies, health stores and supermarkets in the major shopping mall servicing each of the seven districts making up the Durban metropole [Central (The Workshop, City Centre), Outer West (Hillcrest Centre, Hillcrest), South (Galleria Centre, Amanzimtoti), South Central (Chatsworth Centre, Chatsworth), Inner West (The Pavillion, Westville), North Central (Bluff Centre, Bluff) and North (Gateway Centre, Mt. Edgecombe)] and the eight districts making up the Cape Town metropole [South (Blue Route Centre, Tokai), South Peninsula (Long Beach Mall, Fishhoek/Kommetjie), Atlantic Seaboard (Cape Quarter, Greenpoint), City Bowl (V&A, Waterfront), West Coast (Bayside Mall, Bloubergstrand), North (Canal Walk Mall, Century City), Cape Flats (Liberty Promenade, Mitchells Plain) and Helderberg (Waterstone Centre, Somerset West)] were collected (Fig. 1a and b). The following characteristics were captured: formulation (tablet, powder or liquid), composition (strains, number of viable cells/cfu, presence/absence of prebiotics, additional ingredients), target population (general, women, infants), storage (room temperature, refrigerated), price, expiry date, recommended mode of administration, medicinal claims and manufacturers' details.

### Data capture and analysis

Product characteristics were electronically captured in a spread sheet. Statistical analysis was conducted using STATA^TM^ version 12 (StataCorp, College Station, TX, USA). Descriptive measures (mean, median, frequencies and percentages) were used to summarize the data. The shelf life of products was estimated by calculating the time difference between time of survey and expiry date of the product.

## Results

A total of 104 unique probiotic products were surveyed from South Africa (SA), with 94 available in Cape Town and 59 in Durban. Forty-five products were only found in Cape Town, ten were only available in Durban, and 49 products were common to both cities (Fig. 1d). Probiotic range differed by district, with the eight Cape Town districts averaging 37 distinct products per district [highest (64) in the Northern suburbs and the lowest (13) in the Atlantic Seaboard] (Fig. 1a). The seven districts of Durban averaged 16 distinct products per district [highest (30) in the Outer West district to the lowest (5) in the Southern district] (Fig. 1b).

Of the 104 probiotics identified, more than half (51%) had GIT health claims for adults and 17% were indicated for GIT and skin conditions in infants. Less than 10% of the probiotic products had claims in multiple health categories such as GIT and immunological health. Medicinal claims of probiotics in both cities were similar (Fig. 1c). Less than 4% of probiotics in both cities in SA (4/104; including Provacare® Probiotic Vaginal Care, Reuterina™ Femme, UltraFlora® Women’s, Vagiforte® Plus) were explicitly for vaginal health (Fig. 1c).

### Bacterial species included in probiotic formulations

**Fig. 2:**
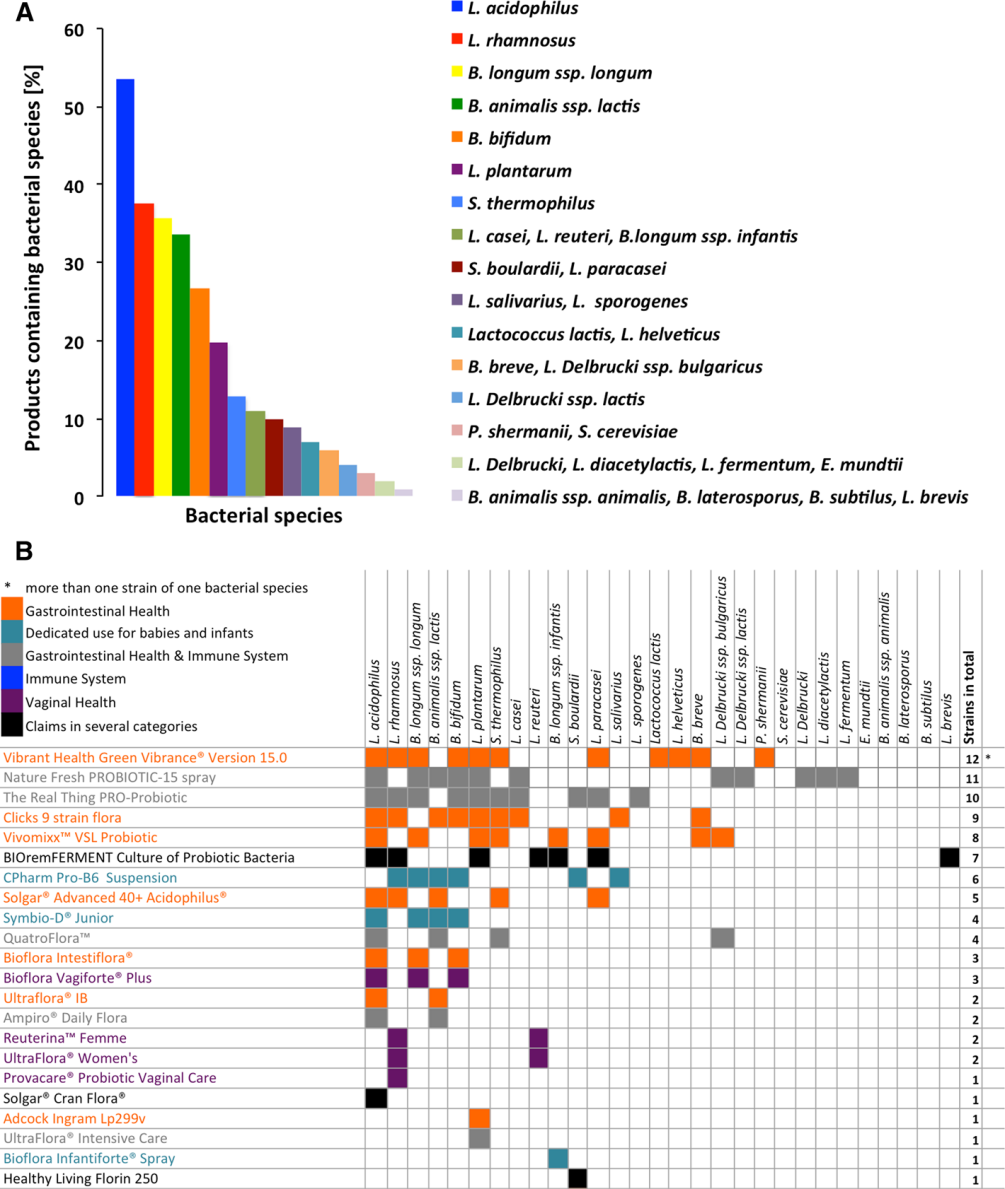
Bacterial distribution in probiotics. **a** Constituting organisms. Bacterial species contained in probiotics belonged to the genera *Lactobacillus* with 15 species*, Bifidobacterium* with 8 species*, Streptococcus thermophilus, Enterococcus mundtii* and *Propionibacterium shermanii*. The yeast *Saccharomyces* was represented with two species. **b** Health claims and association with bacterial composition. Each product was grouped into one health category as described above and bacterial strains were listed. For the complete figure see Additional file 1: Figure S1.1 and Additional file 2: Figure S1.2

The most common bacterial species found in the probiotics belonged to the genera *Lactobacillus* [15 species; most commonly *L. acidophilus* (in 53.5% of the recorded products), *L. rhamnosus* (37.6%), *L. plantarum* (19.8%)] and *Bifidobacterium* [8 species; most commonly *B. longum ssp. longum* (35.6%), *B. animalis ssp. lactis* (33.7%), *B. bifidum* (26.7%)] (Fig. 2a). The most common combinations of species were *L. acidophilus* with *B. longum ssp. longum* (in 24 products), *L. acidophilus* with *B. bifidum* (23 products), *B. longum ssp. longum* with *B. bifidum* (23 products), *L. acidophilus* with *L. rhamnosus* (19 products), and *L. rhamnosus* with *B. longum ssp. longum* (18 products) (Fig. 2b, Additional file 1: Figure S1.1 and Additional file 2: Figure S1.2). Other less common organisms contained in probiotics were *Saccharomyces boulardii* and *S. cerevisiae*, *Streptococcus thermophilus*, *Enterococcus mundtii*, *Lactococcus lactis* and *Propionibacterium shermanii*.

The vaginal probiotics contained either a combination of the three species *L. acidophilus/B. longum ssp. longum/B. bifidum* (Bioflora Vagiforte® Plus), two species *L. rhamnosus/L. reuteri* (Reuterina™ Femme, UltraFlora® Women’s) or the single bacterial species *L. rhamnosus* (Provacare® Probiotic Vaginal Care).

### Dose, formulation and storage

The number of bacterial strains contained per product ranged from 1 (25.0%, 26/104) to 12 (2.9%, 3/104) with a mean of 3. Overall, probiotic products claimed to contain a median of 1.5 × 10^9^ viable organisms ranging from 4 × 10^7^ to 9 × 10^11^ cfu per dose, with daily dosage recommended. The daily dose (capsules per day) of products depended on therapeutic indication: prophylaxis (chronic), treatment (acute) or as an adjunct to antibiotic treatment. However, organisms included in these different probiotic preparations did not differ according to indication. Interestingly, some products formulated with exactly the same bacterial species, strains and cfu were marketed by different manufacturers under both similar and multiple health claims (Additional file 1: Figure S1.1 and Additional file 2: Figure S1.2). Others contained the same bacterial species but with different doses of each species (e.g., two species combination *L. acidophilus*/*B. animalis ssp. lactis*: range 5 × 10^8^ to 15 × 10^9^ cfu or single species *L. sporogenes*: range 4 × 10^7^ to 6 × 10^9^ cfu). In some instances the same manufacturer produced a range of probiotic products with identical bacterial strain content and dose while marketing them as distinct products with different health claims (Additional file 1: Figure S1.1 and Additional file 2: Figure S1.2).

The most common formulation was capsules (59.8%), with some products available as liquids (18.7%), tablets (13.1%) or powder (8.4%). The majority (82.7%, 86/104) were stored on the shelf (room temperature), and 17.3% (18/104) were stored in the fridge (4 °C). Probiotics maintained at 4 °C tended to be from the same manufacturer (10/18 produced by Metagenics^®^ and 5/18 produced by Bioflora CC), but did not appear to be systematically different in formulation, probiotic composition or expiry date. The average shelf life time was 14 months (from time of survey to recorded product expiry date) for both products stored at room temperature and at 4 °C. There was no correlation between shelf life and formulation of the product. Probiotics were predominantly recommended for oral administration (97.1%, 101/104). Of the four vaginal products available, two were intended for oral administration, one was for both oral and vaginal administration and one for vaginal dosing only, and all were stored at room temperature.

Two-thirds (64.4%, 67/104) of the surveyed products were manufactured in SA [88.1% (59/67) of shelf products versus 11.9% (8/67) of refrigerated products]. The total of 104 products were produced by 39 manufacturers, of which 27 (69.2%) were based in SA, mainly in Johannesburg (12/27), Pretoria (6/27) and Cape Town (6/27). Of the four vaginal products, two were manufactured in SA (Reuterina™ Femme, Bioflora Vagiforte® Plus), one in Canada (Provacare® Probiotic Vaginal Care) and one in the US (UltraFlora® Women’s).

Aside from bacteria, most products contained additional ingredients, such as vitamins, minerals, enzymes or fruit extracts. Common enzymes included were digestive enzymes including amylase, protease, invertase, malt diastase, lipase, cellulose or lactase. A few products (8/104, 7.7%) contained folic acid (vitamin B9), which is important for nucleotide synthesis in humans and bacteria but cannot be synthesized by humans. Some bacteria synthesize folate, including many *Bifidobacterium* spp. but only a few *Lactobacillus* spp. and thus, it needs to be ingested [44]. Similar percentages of products contained thiamine (vitamin B1), a coenzyme in the metabolism of sugars and amino acids, and riboflavin (vitamin B2) or nicotinamide (vitamin B3) that are both needed for oxidation-reduction reactions [45]. Those products also included pantothenic acid (vitamin B5), which is a cofactor for the synthesis and metabolism of proteins, carbohydrates and fats, vitamin B6, a coenzyme for amino acid, glucose and lipid metabolism, and biotin (vitamin B7) that is a coenzyme for carboxylase enzymes needed for the synthesis of some amino acids, fatty acids and gluconeogenesis [45].

The majority of products (67.3%) did not contain prebiotics. In those that did, the most common were fructo-oligosaccharides and inulin, which are both indigestible carbohydrates that reach the intestine intact where they encounter *Lactobacillus* and *Bifidobacterium* spp., which are able to metabolize and transport these compounds [46].

### Claims of probiotics for vaginal health

We found four probiotics that made explicit claims on the package insert for vaginal health that were not referenced. None of the four products had MCC or FDA approval and they were not marketed as complementary medicine or as medicines. Reuterina™ Femme claims to help maintain a healthy vaginal flora and prevent urogenital infections and BV. Its dosage can be increased from one to two capsules daily and there are no known symptoms when overdosed, but no references were provided to support this statement. The package insert seems to contradict the claims on the outer packing as it states that this product is not intended to treat, cure or prevent disease. Provacare® Probiotic Vaginal Care is classified as nutritional substance and claims to restore vaginal flora balance and maintain the ideal pH. It also claims to assist in the relief of symptoms of burning, stinging and vaginal discharge. Bioflora Vagiforte® Plus claims to restore and maintain the normal vaginal flora by controlling the overgrowth of pathogenic microorganisms in the GIT and preventing their transfer to the urogenital tract. High dose treatment is stated to prevent the development of BV and candidiasis. The dose is one oral capsule daily for five days and then one tablet vaginally daily for the next five days. UltraFlora® Women’s is taken orally once to twice daily and is claimed to restore and maintain a healthy vaginal microflora, and reportedly reduces pathogenic bacteria and yeasts. Further, UltraFlora® Women’s claims to assist treatment of BV in conjunction with antibiotics when taken twice daily for at least seven days 2–3 h before or after the antibiotics. Those statements have not been evaluated by the FDA.

### Cost of probiotics

The median cost per probiotic capsule or tablet in this study was USD 0.45 [currently equivalent to 6.30 South African Rand (ZAR)], ranging from USD 0.13–3.29 (ZAR 1.80–46.00). Probiotic prices did not differ statistically significantly between Cape Town and Durban (mean of ZAR 6.50 vs. ZAR 6.00), nor when comparing probiotics manufactured locally to those that were imported, or probiotics stored on the shelf to those requiring refrigeration. Instead, prices of products differed according to manufacturer with products from Metagenics^®^, Bioflora CC, Viridian Nutrition and Vibrant Health^®^ being more expensive (mean ZAR 22.40, USD 1.60 per capsule/tablet) when compared to those from other manufacturers evaluated in this study (mean: ZAR 4.60, USD 0.33 per capsule/tablet; *p* < 0.0001). Given that >60% of households of Cape Town and Durban metropoles earned < USD 445.00 (ZAR 6367) per month in 2011 (Census 2011, http://www.statssa.gov.za), the cost of one treatment course per individual of any of the vaginal probiotics publically available in South Africa would use up 1.6–4.9% of monthly income.

## Discussion

The commensal microbiota in the female genital tract is important to protect women against STIs, including HIV. BV is associated with increased risk of infection with HIV [26], other STIs [22, 23], and reproductive complications [19–21]. Management of BV is challenging as it is frequently asymptomatic and thus, the majority of cases go undiagnosed [27]. Further, antibiotic treatment usually fails in the long-term, with recurrence rates of ~30% within three months of treatment and ~50% within six months [29]. As BV is related to an often recurrent deficiency of appropriate commensal microorganisms, adjunctive probiotic therapy could provide significant benefit in ensuring maintenance of a healthy biome in women treated for BV.

The aim of this survey was to determine the availability of vaginal probiotics in South Africa, a region with high BV and HIV burden in young women. We identified 104 products available in Cape Town and Durban, with more products marketed in Cape Town than in Durban. Although there were fewer stores in Durban selling probiotics, these two major cities do not differ in population size, gross domestic product (GDP), average income, unemployment rates or medical aid coverage (http://www.statssa.gov.za), proxies for social-economic status and general health in both cities. Factors contributing to this discrepancy in probiotic availability are likely to be complex, but may include cultural and ethnic differences in the populations, and differences in traditional, complementary and alternative medicine use [47].

The predominant indication for probiotics in South Africa is for GIT health with only four products being marketed for vaginal health, illustrating a huge discrepancy in product availability given BV rates of up to 58% in South Africa [25]. Most of the bacterial strains contained in the four vaginal probiotics identified in this study were not common colonizers of the lower reproductive tract. For instance, *B. longum ssp. longum* and *B. bifidum* are commensal in the GIT [48], while *L. acidophilus, L. reuteri and L. rhamnosus* primarily colonize the GIT [49], but also have been isolated from the female genital tract [41].

While the efficacy of probiotics still needs to be proven in clinical trials, the development of vaginal probiotics should, as a reasonable starting point, contain species that are frequently commensals of the healthy vaginal tract, such as *L. crispatus, L. gasseri, L. jensenii, L. vaginalis* and *L. iners*. The efficacy of probiotic combinations of vaginal commensal bacterial species, strains and dose along with adjunctive antibiotic use to treat BV and prevent its recurrence needs also to be evaluated.

The most common bacterial species found overall in probiotics in the South African market were *L. acidophilus*, *L. rhamnosus*, *B. longum ssp. longum*, *B. animalis ssp. lactis*, *B. bifidum* and *L. plantarum*. Some reasons cited for the use of *L. acidophilus* strains in probiotic formulations include that it is stable in products, resistant to GIT bile, tolerant to low pH, and adherent to human colonocytes in cell culture [50–53]. In addition, they produce antimicrobial substances and contain lactase activity, meeting the criteria needed for an effective probiotic [54]. Similar characteristics have been described for all the above-mentioned *Lactobacillus* and *Bifidobacterium* spp. commonly found in probiotics [55–57]. Additionally, these probiotic strains do not compete with each other for essential nutrients and therefore, can be combined in products [56]. *Bifidobacterium* spp. are able to produce acetic acid that reduces yeast growth [58], which may be a reason why it is contained in some vaginal probiotics, although these species are much less common in the female genital tract than *Lactobacillus* spp. [59].

In addition to *Lactobacillus* and *Bifidobacterium* spp., other species were less commonly found in probiotics in SA, including *S. boulardii, S. cerevisiae* and *E. mundtii*. The non-pathogenic yeast strains *S. boulardii* and *S. cerevisiae* may regulate intestinal microbial homeostasis [60], interfere with the ability of pathogens to colonize and infect the mucosa [61–63], modulate immune responses [64–66], stabilize the gastrointestinal barrier function and induce absorption of nutrients [67]. The inclusion of *E. mundtii* in probiotics is controversial as it is thought to be marginally virulent, with reports of endophthalmitis (inflammation of the intraocular cavities) published [68, 69] and it is not considered safe by the FDA [69].

In the USA, the FDA applies a complex framework of regulation to validate manufacturers’ claims for the products they market [70]. In this framework, a health claim is “any claim made on the label or in labelling of a food, including a dietary supplement, that expressly or by implication, including ‘third party’ references, written statements, symbols, or vignettes, characterizes the relationship of any substance to a disease or health related condition” [71]. In the case of vaginal probiotics, this could possibly be a reduction in the risk of incurring BV in a healthy population. While regulatory bodies (including the FDA and the South African MCC) are more concerned with product safety than misleading claims, sound scientific approaches need to be used to demonstrate the health benefits of probiotics such as dose-response relationship [72]. Previous studies have shown poor correlation between label claims and actual probiotics content [73, 74], so post-market surveillance should be mandatory to demonstrate health benefits related to probiotics.

This survey had some limitations. The exact shelf lifetime could not be determined because only the expiry date and date of survey, but not the date of production were captured. We did not look at any other cities in the country but restricted our study to two of the larger cities, and only looked at one centre per district within these cities.

## Conclusions

This study provides a strong rationale for the development and clinical evaluation of additional probiotics for vaginal health, as these products are underrepresented in the South African probiotic market. Adjunctive therapy with probiotics targeting young women with BV in regions with highest BV and HIV incidence has the potential to provide considerable benefit. A cost analysis of the products in our study confirmed that the prices of these probiotics still represent a considerable barrier in their limited uptake in the segments of the population most in need. This highlights the need for the development and testing of cheaper, alternative products, which will have the added benefit of being tailor-made for the South African population. The increased availability of vaginal probiotics represents an intervention with significant potential that can help decrease the burden of BV in the South African community.

## Additional files

Additional file 1: Figure S1.1. Products, their health claim and contained bacterial species. (TIFF 1023 kb)
Additional file 2: Figure S1.2. Products, their health claim and contained bacterial species. (TIFF 944 kb)

## Abbreviations

BV: Bacterial vaginosis
cfu: Colony-forming units
FDA: Food and Drug Administration
GDP: Gross domestic product
GI: Gastrointestinal
GIT: Gastrointestinal tract
MCC: Medicines Control Council
SA: South Africa
SSA: Sub-Saharan Africa
STI: Sexually transmitted infections
