# Supplementary figures and images for: Probiotics for vaginal health in South Africa: what is on retailers’ shelves?
Source: BMC Womens Health. 2017 Jan 19;17:7. doi: 10.1186/s12905-017-0362-6 (PMC5248517; doi:10.1186/s12905-017-0362-6)

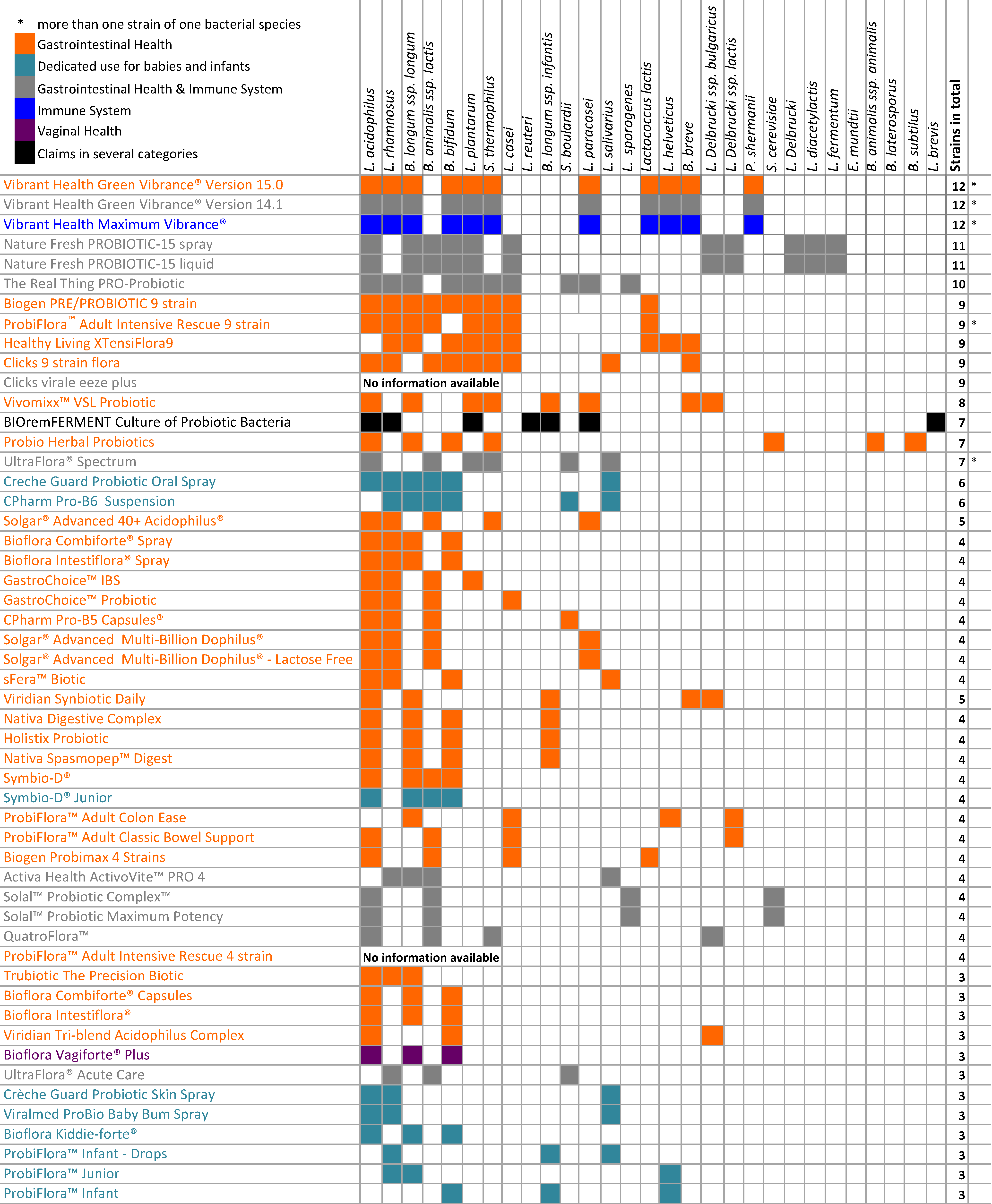

Supplement: Additional file 1: Figure S1.1. — Products, their health claim and contained bacterial species. (TIFF 1023 kb) [file 12905_2017_362_MOESM1_ESM.tiff]

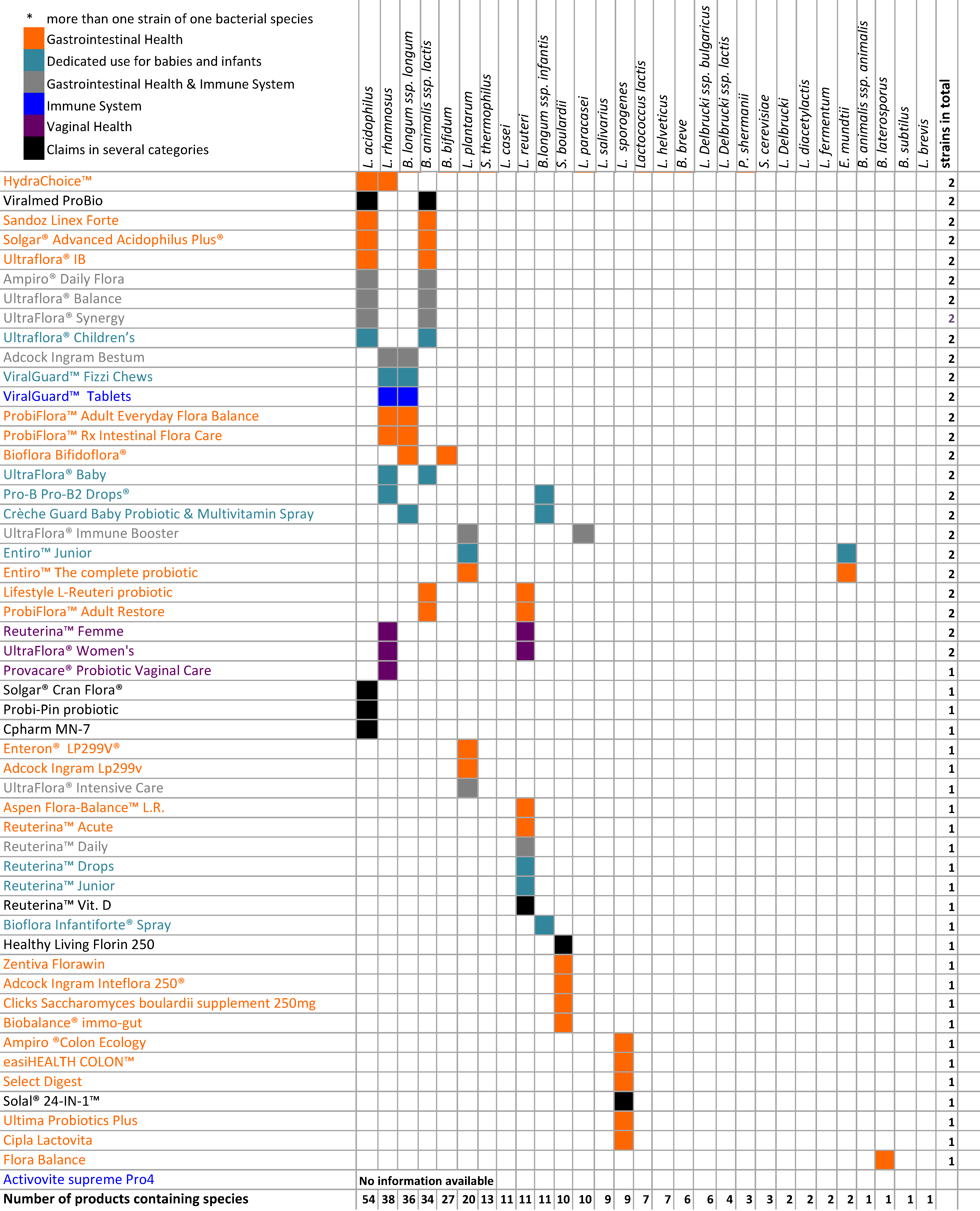

Supplement: Additional file 2: Figure S1.2. — Products, their health claim and contained bacterial species. (TIFF 944 kb) [file 12905_2017_362_MOESM2_ESM.tiff]
